# Fixation of Expression Divergences by Natural Selection in *Arabidopsis* Coding Genes

**DOI:** 10.3390/ijms252413710

**Published:** 2024-12-22

**Authors:** Cheng Qi, Qiang Wei, Yuting Ye, Jing Liu, Guishuang Li, Jane W. Liang, Haiyan Huang, Guang Wu

**Affiliations:** 1 College of Life Science, Shaanxi Normal University, Xi’an 710119, China; dancing@snnu.edu.cn (C.Q.); yeyuting123@foxmail.com (Y.Y.); jingliu@snnu.edu.cn (J.L.); guishuangli@snnu.edu.cn (G.L.); 2 Department of Statistics, University of California, Berkeley, CA 94720, USA; jwliang@stanford.edu (J.W.L.); hyh0110@berkeley.edu (H.H.)

**Keywords:** functional divergences, gene expression divergences, natural selection, protein-coding genes, *Arabidopsis*

## Abstract

Functional divergences of coding genes can be caused by divergences in their coding sequences and expression. However, whether and how expression divergences and coding sequence divergences coevolve is not clear. Gene expression divergences in differentiated cells and tissues recapitulate developmental models within a species, while gene expression divergences between analogous cells and tissues resemble traditional phylogenies in different species, suggesting that gene expression divergences are molecular traits that can be used for evolutionary studies. Using transcriptomes and evolutionary proxies to study gene expression divergences among differentiated cells and tissues in *Arabidopsis*, expression divergences of coding genes are shown to be strongly anti-correlated with phylostrata (gene ages), indicators of selective constraint Ka/Ks (nonsynonymous replacement rate/synonymous substitution rate) and indicators of positive selection (frequency of loci with Ka/Ks > 1), but only weakly or not correlated with indicators of neutral selection (Ks). Our results thus suggest that expression divergences largely coevolve with coding sequence divergences, suggesting that expression divergences of coding genes are selectively fixed by natural selection but not neutral selection, which provides a molecular framework for trait diversification, functional adaptation and speciation. Our findings therefore support that positive selection rather than negative or neutral selection is a major driver for the origin and evolution of *Arabidopsis* genes, supporting the Darwinian theory at molecular levels.

## 1. Introduction

Functionalization of preexistent genes by divergences in their coding sequences and regulatory sequences (expression) is a major mechanism to generate trait variation [1,2,3,4,5,6,7]. It has been shown that divergences in gene expression have less detrimental effects on fitness, likely allowing greater flexibility for the fixation of such divergences, leading to trait variation, adaptation and speciation [8,9,10,11,12]. Therefore, gene expression divergences may explain even more phenotypic divergences than protein-coding sequence divergences can during evolution [8,9,13]. However, complex biological traits are gradually refined by the integration of divergences in gene coding sequences and expression over time. Thus, it is of great interest to study the underlying genetic mechanism of gene expression divergences, or how divergences in gene coding sequences and expression have been integrated to refine biological traits and characters during evolution [2,9,14,15,16,17]. However, the dynamics of gene expression evolution at the genome scale are still poorly understood.

Darwinian theory postulates that evolution is a slow and gradual process, and that speciation is the result of many small changes that accumulate over long periods of time, leaving molecular imprints accumulated in extant species [18,19]. Thus, it is possible to use related extant species to evaluate the evolutionary processes of numerous biological traits and characters. One common practice is to compare either biological trait and character divergences between differentiated cells, tissues and organs in the same species, or biological trait and character divergences between analogous cells, tissues and organs in different species [2,8,14,20]. Divergences in expression (differential gene expression) are quantifiable traits that bridge static genotypic information and dynamic biological traits and characters, which can recapitulate known developmental models in the same species or traditional phylogenies among different species [8,21,22,23,24]. Thus, differential gene expression patterns can be a convenient molecular trait to study evolutionary mechanisms [8,9,25,26,27,28,29,30,31,32,33,34,35]. Although both adaptive selection and neutral selection have been implicated in shaping differential gene expression [8,9,16,25,26,27,28,29,30,31,32,33,34,35], the current conclusion is that the relaxation of selective constraints by neutral selection, together with purifying selection, is considered the major cause for the evolution of gene expression divergences [21,22,23,24,36,37]. This is contradictory to the Darwinian theory that emphasizes the role of positive selection for trait evolution and speciation, creating a paradox that needs to be addressed [38,39].

For coding genes, genetic divergences can be partitioned into nonsynonymous replacements (nucleotide changes resulting in amino acid changes) and synonymous substitutions (nucleotide changes resulting in no amino acid changes) [40]. Ka is the ratio of the number of nonsynonymous replacements per nonsynonymous site, while Ks is the number of synonymous substitutions per synonymous site [41]. Ka is presumably subject to natural selection, while Ks, which is generally considered to reflect the neutral nucleotide substitution rate, is not [41]. By comparing the ratio of Ka to Ks, the extent of selection involved in nucleotide substitutions can be determined [41]. Thus, Ka/Ks (ω) can be used as an indicator of selective pressure (selective constraint) acting on a protein-coding gene [41]. Hence, Ks and ω can be used as evolutionary proxies to define sequence divergences in the coding genes (especially for short-term evolution) and to study the evolutionary mechanism of gene expression divergences (Table S1) [23,24,36,42,43]. In the context of expression-based evolutionary analysis, gene expression abundance (GEA), which reflects quantitative aspects of gene expression, and gene expression breadth (GEB), which represents specific patterns of gene expression in a spatial and temporal manner, are usually used as indicators [44].

To clarify that GEA and GEB are indeed the most significant correlates associated with evolutionary rates in multicellular organisms, this study used EST (expressed sequence tag) and cDNA collections (https://www.arabidopsis.org/download/list?dir=Genes%2FTAIR10_genome_release%2FTAIR10_gene_transcript_associations, accessed on 20 December 2020), RNA-Seq and microarray data to assess gene expression across the genome of *Arabidopsis thaliana* (Table S1) [23,24,29,45,46,47]. Furthermore, the phylostratum (PS) was incorporated as a long-term evolutionary proxy that reflects gene ages (Table S1) [23,24,40] to study the evolutionary mechanism of gene expression divergences in *A. thaliana*. The model plant *A. thaliana* and its relative *A. lyrata* are thought to have derived from a common ancestor 5~13 million years ago and share 85% nucleotide identity in two genomes (93% for protein-encoding genes) [10,48]. However, *A. thaliana* is an annual self-fertilizing plant, while *A. lyrata* is a perennial outcrossing species with self-incompatibility [49]. The substantial phenotypic and genomic diversification of these two closely related species suggests that significant biological changes (divergences) have been adapted since the split from their last common ancestor, providing abundant genetic variations to study the evolutionary mechanisms of expression divergences of the coding genes in *A. thaliana.* This study used *A. thaliana* Col-0 and *C. rubella* (intergenus), *A. thaliana* Col-0 and *A. lyrata* (interspecies) and *A. thaliana Col-0 and Ws* (intraspecies) as materials to systematically compare GEA and GEB on their correlation with PS and evolutionary rates using EST, cDNA, RNA-Seq and microarray datasets. The findings from this study have the potential to enhance the understanding of the factors influencing the evolutionary rates of proteins in multicellular organisms, so as to provide some evidence for the controversy of whether gene expression divergence is mainly driven by neutral selection or Darwinian natural selection.

## 2. Results

### 2.1. Gene Expression Abundance (GEA) Was Anti-Correlated with Phylostratum (PS)

**Figure 1:**
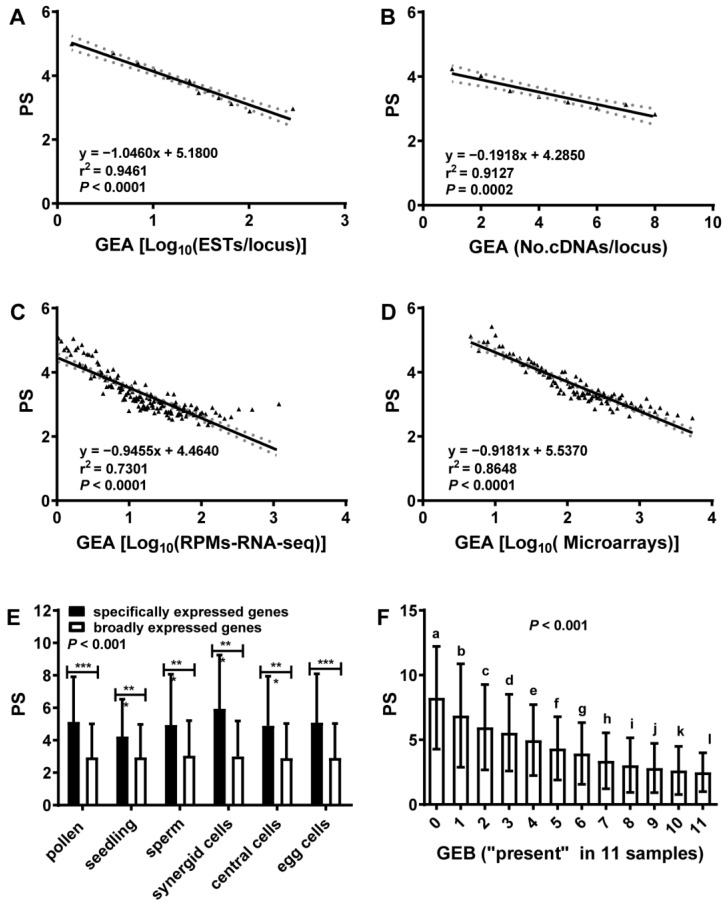
Gene expression abundance (GEA) was anti-correlated with phylostratum (PS). (**A**) ESTs/locus was strongly anti-correlated with PS. (**B**) cDNAs/locus was strongly anti-correlated with PS. (**C**) Log_10_(RPMs) from RNA-Seq of seedlings was strongly anti-correlated with PS. Each data point is an average of 100 loci grouped by expression amount. RPMs: reads per million. (**D**) Log_10_(microarray signals) from microarray data [36] was strongly anti-correlated with PS. Each data point was an average of 100 loci grouped by expression amount. (**E**) Genes expressed in one sample (black bars) had a higher PS than genes expressed in more than one sample (unfilled bars). *, **, and *** indicate significant differences at *p* < 0.05, *p* < 0.01, and *p* < 0.001 (*t*-test), respectively. (**F**) Genes with smaller GEB (narrow expression) had a higher PS than genes with larger GEB (broad expression). Letters indicate significant differences at *p* < 0.001 (One-way ANOVA). Grey lines indicate 95% confidence intervals and triangles represent data points (**A**–**D**). Error bars are standard deviation (**E**,**F**).

This study assigned all *Arabidopsis* genes to a PS (from 1 to 13) to coalesce a gene clade based on the origin of a defined protein domain in organisms during evolution, as shown in Refs. [23,50,51] and Table S1. PS1 was defined for the most ancient genes originating in single-celled organisms and PS13 for the youngest genes specific to *A. thaliana* (Table S1). Therefore, the younger the gene, the bigger the PS. To analyze GEA, we counted the number of ESTs or cDNAs per locus (Tables S2 and S3) and assigned each data point by averaging 100 loci, first sorted by gene expression amount in microarray or RNA-Seq datasets (Tables S4 and S5). These results showed that GEA was anti-correlated with PS (Figure 1A–D and Tables S2–S5), suggesting that new genes have lower expression levels. Additionally, the genes specifically expressed in a sample (specific expression) had a significantly higher PS than genes expressed in more than one sample (broad expression) (*p* < 0.001) (Figure 1E and Table S6). To further analyze GEB, we counted the “present call” in samples with expression data in specific cell types, such as single-celled sperm, synergids, central cells, eggs and three-celled pollen, as well as multi-celled samples. For EST or cDNA datasets, the “present call” meant that the locus had at least one EST or cDNA sequence recorded in the collections, and for microarray or RNA-Seq datasets, the “present call”, as the output of the AtPANP program, meant that each locus had a signal beyond a defined signal threshold (see methods) (Table S7) [29,47,52,53]. Figure 1F showed that PS was anti-correlated with GEB, which was obtained by counting the numbers of “present call” in 11 samples, indicating that new genes had narrower GEB (higher gene expression specificity) (Table S7). Together, these results suggest that the overall evolutionary trend for gene expression is to evolve from high to low expression levels and from broad to specific tissues and organs, thereby increasing the expression divergences over time.

### 2.2. Evolutionary Analysis and Identification of Loci with Positive Selection (ω > 1) in Arabidopsis

To test the approximation of normal distribution at a more relaxed but still informative resolution level, the data was partitioned into 30 equal quantile intervals. In this study, the Ks and Ka/Ks (ω) were obtained for 20,729 orthologous gene pairs between *A. thaliana* and *A. lyrata* (between species) (Tables S1 and S8), and the results showed that Ks exhibited a nearly normal distribution while ω did not (Figure S1A,B). This suggests that Ks from orthologous gene pairs between species (At-Al) can be regarded as markers for neutral selection, and that the selective constraint ω can be regarded as standardized Ka for each individual locus. Additionally, Ks and ω were obtained from 18,056 and 8005 orthologous gene pairs between *A. thaliana* and *C. rubella* (between genera), and between *A. thaliana* ecotypes (within species). Ks between genera (At-Cr) was close to normal distribution, but Ks within species (Ws-Col) was not (Figure S1C,E), supporting mutational bias [54]. Yet, both Figure S1A,C were “reasonably approximated” by a normal distribution (*p*-values were the same at 0.2566 for both Figure S1A,C). Consistent with nonrandom selection on ω between species, there were skewed distributions for ω in orthologous gene pairs between genera or within species (Figure S1D,F). These approximations demonstrated that Ks between species and genera were reasonably close to being unselected (neutral selection).

To further evaluate the genomic effect on protein evolution in *Arabidopsis* genes, the distribution and relationship of Ks and ω in the genome of *A. thaliana* were analyzed. Running with the code in “normal.test.r” (R version 4.0.1, R Foundation for Statistical Computing, Vienna, Austria, https://cran.r-project.org/src/base/R-4/, accessed on 6 June 2021), the locus from orthologous gene pairs between species/genera and within species (At-Al/At-Cr/Ws-Col) were evaluated and normalized. Roughly, 80% of the *A. thaliana* genes (20,729/27,206) were analyzed, and the percentage of genes analyzed on each chromosome was also close to 80%. The distribution of loci used in our analysis was also similar to the distribution of all loci in each chromosome, and also between genera (18,056/27,206) and within species (8005/27,206) (Tables S1 and S8). Therefore, there was no clear bias for loci selected for ω analysis. The ω for loci across all chromosomes was then analyzed. We determined the mean ω of all 20,729 (18,056, 8005) loci between species (between genus and intraspecies), with the smallest in between species and the largest in between genera, while Ks was the opposite, supporting that new(er) genes evolved the fastest. The averages of Ks and ω on each chromosome were very similar to the average of ω across the genome (Tables S1 and S8), and ω between any two chromosomes exhibited no significant difference (Table S8). The locus of ω > 1 is considered to be a positive selection site. The distribution of ω > 1 loci between species (between genera, within species) was similar to the distribution of all loci in each chromosome as well. Therefore, there was no clear bias for loci selected for ω > 1 analysis (Table S8).

**Figure 2:**
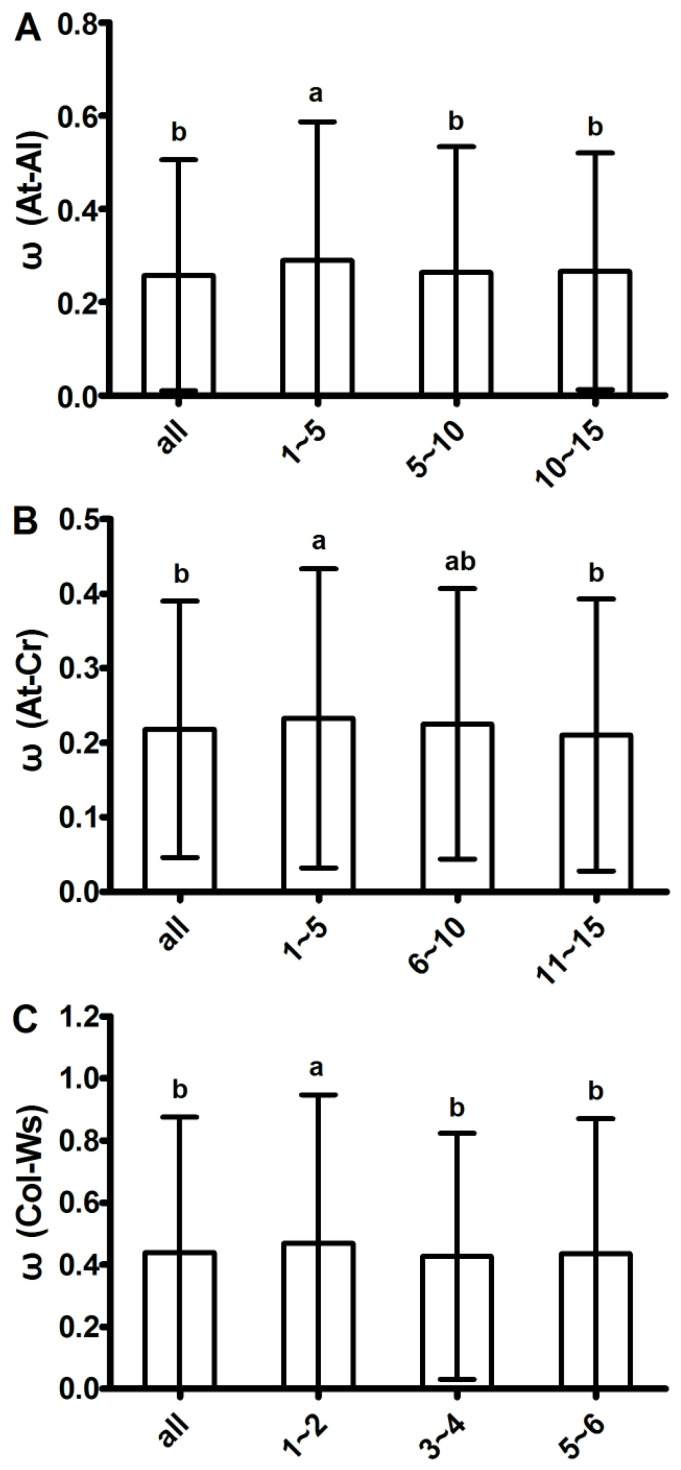
Linkage disequilibrium near positive selection (ω > 1) loci in *Arabidopsis*. Linkage disequilibrium near ω > 1 loci derived from orthologous gene pairs between *A. thaliana* and *A. lyrata* (interspecies; (**A**)) and between *A. thaliana* and *C. rubella* (intergenus; (**B**)). The 0 represents ω > 1 loci; 1–5 represents loci closest to locus 0 (5 on each side), while 6–10 and 11–15 represent positions of loci farther away from locus 0, with the distribution of ω for loci in each group (all loci, 1–5, 6–10 and 11–15, respectively). (**C**) Linkage disequilibrium near ω > 1 loci from orthologous gene pairs within *Arabidopsis* species. The 0 represents ω > 1 loci; 1–2 represents loci closest to locus 0 (2 on each side), while 3–4 and 5–6 represent positions of loci farther away from locus 0, distribution of ω for loci in each group (all loci, 1–2, 3–4 and 5–6, respectively). Letters indicate significant differences at *p* < 0.05 (*t*-test). Error bars are standard deviation.

It was suspected that the non-normal distribution of ω was due to hitchhiking by positive selection. To address this issue, loci with positive selection were searched and 94, 341 and 630 loci with ω > 1 in orthologous gene pairs between genera, between species and within *A. thaliana* were found, respectively. Examining the loci flanking these ω > 1 loci using a linkage disequilibrium test showed that ω was significantly higher for loci adjacent to ω > 1 loci than for loci further away from ω > 1 loci (Figure 2 and Table S9). Together, our results suggest that these ω > 1 loci may represent bona fide positive selection, and that there are more positively selected loci in orthologous gene pairs within species than between species or genera, which heightened ω with a skewed normal distribution in *Arabidopsis*.

### 2.3. Gene Expression Abundance (GEA) Was Anti-Correlated with Selective Constraint (ω)

**Figure 3:**
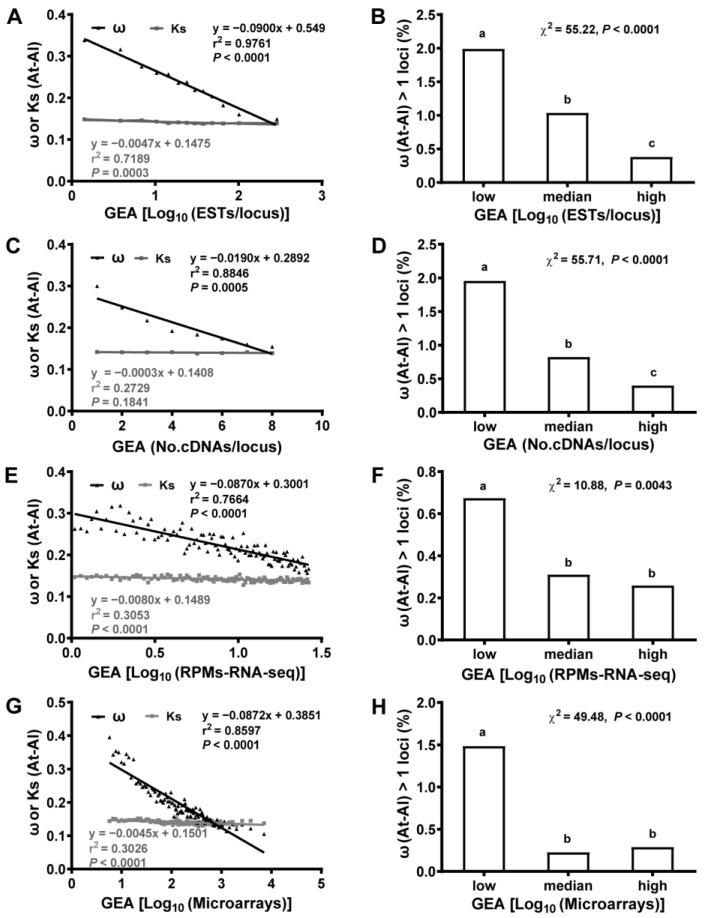
Gene expression abundance (GEA) was strongly anti-correlated with selective constraint (ω) and the incidence of ω > 1 loci (positive selection markers) derived from orthologous gene pairs between *A. thaliana* and *A. lyrata* (interspecies). GEA for ESTs, cDNAs, microarray and RNA-Seq data was treated as described in the main text and methods, as well as in Tables S10–S13 (**A**,**C**,**E**,**G**), while GEA in (**B**,**D**,**F**,**H**) was divided into low, medium and high levels and then correlated with ω > 1 loci. ESTs/locus was anti-correlated strongly with ω (**A**) and the incidence of positive selection (**B**) but weakly correlated with Ks (**A**). cDNAs/locus was anti-correlated only with ω (**C**) and the incidence of ω > 1 loci (**D**) but not with Ks (**C**). Log_10_(RPMs) from RNA-Seq of seedlings was strongly anti-correlated with ω (**E**) and the incidence of ω > 1 loci (**F**) but correlated weakly with Ks (**E**). RPMs: reads per million. Log_10_(microarray signals) from microarray data [36] was strongly anti-correlated with ω (**G**) and the incidence of ω > 1 loci (**H**) but weakly correlated with Ks (**G**). Each data point was an average of 100 loci grouped by expression amount (**E**,**G**). For (**B**,**D**,**F**,**H**), the letters indicated the significant difference detected by χ^2^ test (*p* < 0.05).

This study next correlated these short-term evolutionary proxies (Ks and ω) with gene expression abundance (GEA), such as ESTs/locus, cDNAs/locus, microarray data from multiple samples and RNA-Seq data from seedlings. There was strong anti-correlation between GEA and ω (Figure 3A,C,E,G and Figure S2A,C,E,G, Tables S10–S13). However, Ks, which represents neutral selection, had almost no correlation with expression levels derived from ESTs, cDNAs, microarrays or RNA-Seq approaches (Figure 3A,C,E,G and Figure S2A,C,E,G and Tables S10–S13). Indeed, loci were divided into three types (low/medium/high) according to their expression abundance (GEA) [21,26], and the lower abundance loci had the more ω > 1 loci (positive selection), which suggested a faster rate of gene evolution in them, the more genes were positively selected [13,55]. There was strong anti-correlation of GEA derived from expressions found in ESTs, cDNAs, microarrays and RNA-Seq datasets with the incidence of ω > 1 loci (positive selection) (Figure 3B,D,F,H, and Tables S10–S13). Similar results were obtained when we correlated GEA with Ks, ω and the incidence of ω > 1 loci derived from orthologous gene pairs between genera (Figures S3 and S4, and Tables S14–S17). However, we did not observe similar levels of significant differences for correlations of GEA with Ks, ω and the incidence of ω > 1 loci derived from orthologous gene pairs within species (Figures S5 and S6, and Tables S18–S21), consistent with a skewed distribution of Ks within species. This is likely due to a broad presence of positive selection (Figure S1). Still, statistically, the correlations of GEA with ω were significantly different from the correlations of GEA with Ks in all but one: the correlations of GEA derived from cDNAs with ω and Ks, in pairwise comparison (Figure S5C, Tables S19 and S22, and method). Together, our results implicate that the genes with faster evolution have a lower expression abundance, and the genes with slower evolution have a higher expression abundance, which is especially related to ω and the positive selection site of ω > 1, but not the Ks of the neutral mutation.

### 2.4. Gene Expression Breadth (GEB) Was Strongly Anti-Correlated with Selective Constraint (ω)

Because of the complex nature of the ESTs, cDNAs, microarray and RNA-Seq data used, the anti-correlation of GEA with ω and the incidence of positive selection can also be interpreted as a correlation of GEB with ω and the incidence of positive selection. This is since lower GEA could be due to high expression in a few cells or cell types, or low expression in many cells or cell types [13], such as the female gametophyte comprised of an egg, a central cell, two synergids and three antipodal cells [56]. The differentiation of these and other specific cell types, tissues or organs is presumably a result of differential gene expression [29]. Therefore, we compared the evolutionary rate (ω) of genes that are only expressed in specific organs and genes that are broadly expressed. The significance analysis showed that genes specifically expressed in one sample had significantly higher ω than genes expressed in more than one sample (*p* < 0.001), with the highest ω in samples with a single cell type (Figure S7 and Tables S23–S25), but Ks had no significant difference between them. In addition, the genes only expressed in samples with one cell type (i.e., sperm, synergids, central cells or eggs) had higher ω than genes only expressed in samples with more than one cell type (i.e., seedlings, pollen) (Figure S7 and Tables S23–S25). Furthermore, female-enriched genes had significantly higher ω than the average of all loci in *A. thaliana* (Table S26). These findings implied a faster evolutionary rate for genes that are specifically expressed rather than those that are broadly expressed.

**Figure 4:**
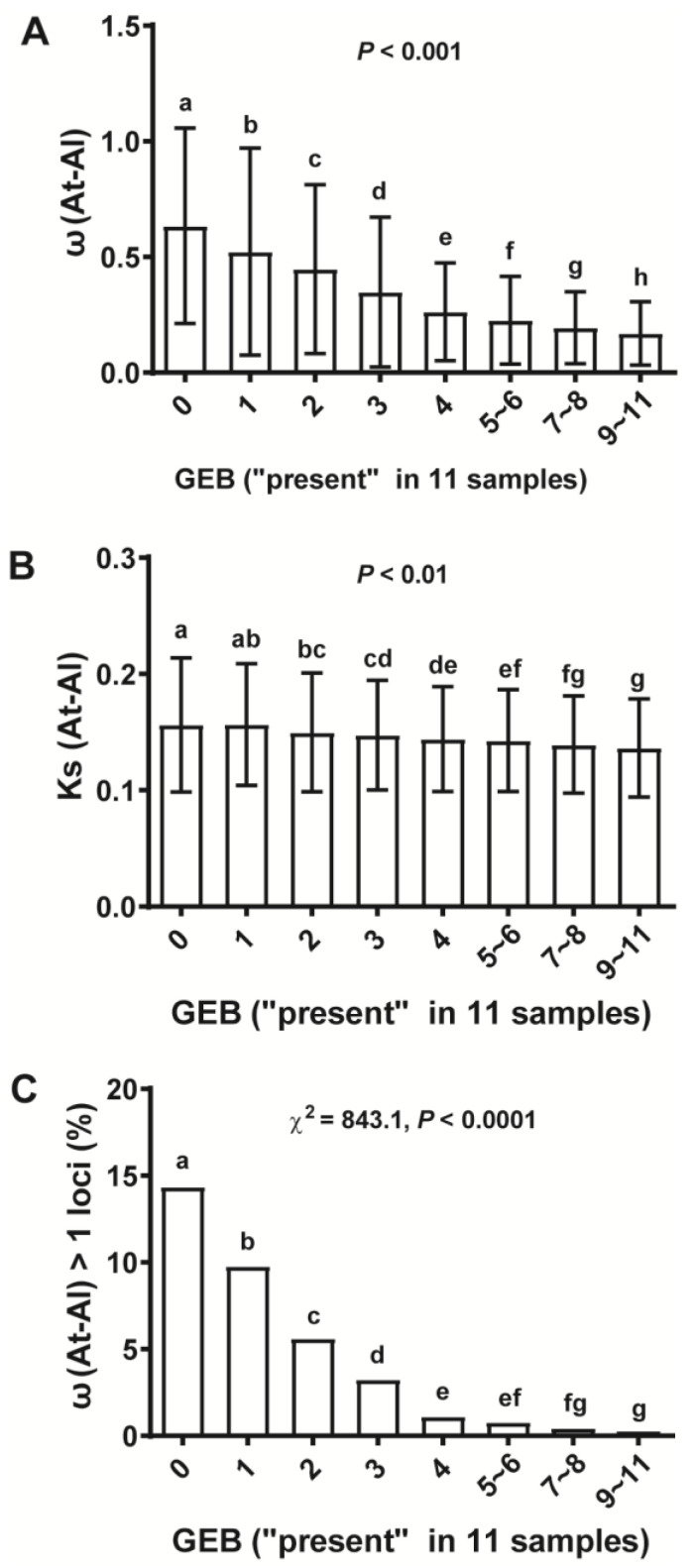
Gene expression breadth (GEB) was strongly anti-correlated with selective constraint (ω) and the incidence of ω > 1 loci (positive selection markers) derived from orthologous gene pairs between *A. thaliana* and *A. lyrata* (interspecies). (**A**) GEB was strongly anti-correlated with ω. Letters indicate significant differences at *p* < 0.001 (One-way ANOVA) for ω between any adjacent groups with differential GEB. Error bars are standard deviation. (**B**) GEB was only minimally anti-correlated with Ks (neutral selection markers). Letters indicate significant differences at *p* < 0.01 (One-way ANOVA). There was no significant difference for Ks between any adjacent groups. Error bars are standard deviation. (**C**) GEB was strongly anti-correlated with the incidence of ω > 1 loci (positive selection markers). Narrowly expressed genes had a significantly higher incidence of ω > 1 loci than did broadly expressed genes. The parameter is the result of a chi-squared test between the levels of GEB and whether the gene is under positive selection (χ^2^ = 843.1, *p* < 0.0001). Error bars are standard deviation (**A**,**B**).

To further evaluate this hypothesis, GEB was applied by counting presence/absence in transcriptomes derived from ESTs, cDNAs and microarray and RNA-Seq data. Analysis of a collection of 11 samples from different plant organs showed that ω decreased with increasing GEB; thus, there was a negative correlation between GEB and ω (Figure 4A) but not GEB and Ks (Figure 4B). The genes with differential GEB had significantly different ω, with the highest ω values for genes with no expression or with expression in only one sample with the highest ω values (specific expression) (Figure 4A and Table S27). This was followed by investigating the relationship of GEB and the presence of neutral selection and positive selection, which showed a significantly higher incidence of ω > 1 loci in groups of genes with smaller GEB than in groups of genes with larger GEB (Figure 4C). There was a weak anti-correlation of GEB and Ks (Figure 4B), consistent with the idea that some synonymous nucleotides are adaptive [57]. Similar results were obtained for correlations of GEB with ω, Ks and incidence of ω > 1 loci derived from orthologous gene pairs between genera and within species, respectively (Figures S8 and S9, Tables S28 and S29), although the correlation trends between GEB and Ks or ω within species were weaker than the trends between interspecies and intergenus (Figure 4, Figures S8 and S9). Together, our results strongly support the notion that positive selection is a driver for the evolution of GEB divergences.

### 2.5. Functional Differences Between Gene Age and Gene Expression Patterns Supported by Mutant Analysis in Arabidopsis

To further support our findings, this study examined distinct expression patterns among representative genes from different PS, including new and ancient genes. Table 1 shows the phylogenetic distribution, with ancient genes AT2G13560, AT2G33210 and AT5G02870 (PS1) representing early-evolved genes, and AT3G18980 and AT2G28240 (PS11) representing a more recently evolved gene. Analysis of *Arabidopsis* expression data revealed that the ancient genes AT2G13560, AT2G33210 and AT5G02870 consistently exhibited the highest expression levels among analyzed genes (Table 1). The recently evolved AT3G18980 and AT2G28240 exhibited minimal GEA (Table 1). Consistently, the previously reported mutant characterization provided additional supports for these observations. These expression patterns and pieces of functional evidence strongly support the speculation that phylogenetically ancient genes (i.e., PS1) typically exhibited higher GEA and broader GEB as well as a broader function, whereas evolutionarily younger genes (i.e., PS11) demonstrated reduced GEA and increased tissue specificity and functional specificity.

### 2.6. Functional Enrichments of Putative ω >1 Loci in Arabidopsis

**Figure 5:**
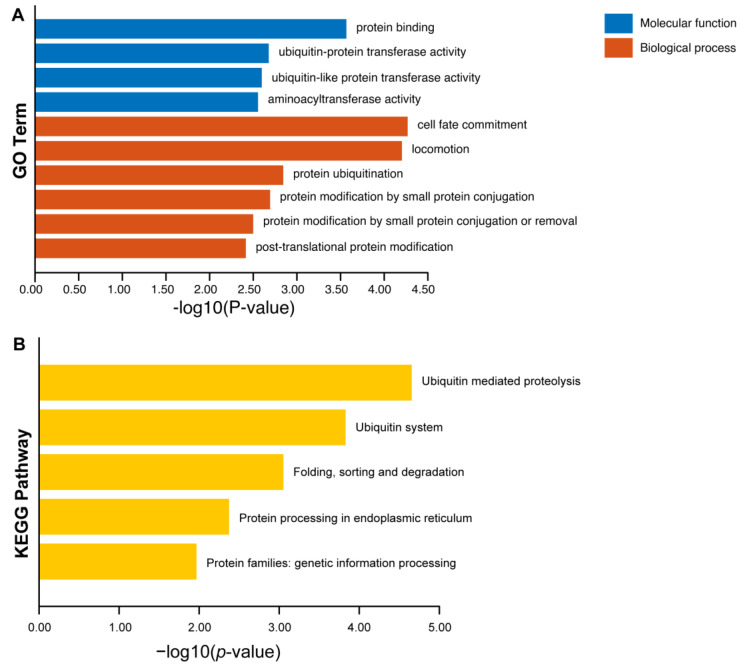
Functional enrichment analysis of putative ω >1 loci in *Arabidopsis*. (**A**) GO enrichment analysis of putative positively selected genes. (**B**) KEGG enrichment analysis of putative positively selected genes. Yellow strips represent the enrichment score [−log10(*p*-value)] of the pathway. Significantly enriched KEGG pathways (*p* < 0.05) are presented.

To further investigate the functional enrichments of the putative ω >1 loci in *Arabidopsis*, gene ontology (GO) and Kyoto Encyclopedia of Genes and Genomes (KEGG) analyses were performed using TBtools v2.142 [63]. A total of 55 genes from 226 putative positively selected genes were used in GO and KEGG enrichment analysis, and others were putative genes without functional annotations (Table S1K). The results of GO enrichment revealed that the predominant biological processes were associated with cell fate commitment, locomotion and protein modification processes (Figure 5). Furthermore, they were classified into different functional categories according to the GO term enrichment analysis, and the key subcategories of molecular function included catalytic activity and protein binding (Figure 5). The KEGG enrichment analysis of 48 genes from 226 putative positively selected genes showed that these genes might be involved in post-translational modifications, i.e., the ubiquitin-mediated proteolysis pathway and protein processing pathway (Figure 5, Table S1K).

## 3. Discussion

Phenotypic variation between or within species can be caused by divergences in protein sequences or gene expression, or both [1,2,3,4,5,6,7]. Changes in coding regions, regulatory elements and epigenetic modifications are all relevant to gene expression [34,64,65,66,67,68,69]. Thus, understanding how sequence and expression divergences of coding genes integrate to control trait evolution over time is a key question to be addressed [3,27,67,68,69,70]. Previous reports have suggested that gene expression abundance (GEA) positively correlated with gene expression breadth (GEB) [44,71]. However, only GEB negatively correlated with the evolutionary rate between human and mouse genes [44]. Additionally, some studies proposed that relaxation of purifying selection by mutations causes rapid evolution [8,30,31,33,34]. If so, Ks should be correlated with GEA, as was ω. This is supported by several studies using unicellular organisms (i.e., *Saccharomyces cerevisiae*) [72]. However, in this study, specific and low gene expression were closely correlated with new genes with high ω, and the high incidence of loci with positive selection but only weakly or not correlated with Ks [13], consistent with the idea that some synonymous nucleotides are adaptive [57]. Our findings thus support that positive selection rather than negative or neutral selection is a major driver for the origin and evolution of the *Arabidopsis* genes.

Our results, alongside numerous reports [8,9,30,31,32,33,34], suggest that higher GEA and larger GEB are correlated with strong purifying selection, suggesting that purifying selection is a key factor for the conservation of gene expression. However, if purifying selection dictates the evolutionary direction of gene expression, the overall evolutionary trend for gene expression should be from specific and low to broad and high. However, from unicellular ancestors to multicellular organisms, speciation requires genes to be differentially and specifically expressed [35], suggesting that gene expression should have evolved from broad and high to specific and low. By integrating gene age and expression profiles, we found that the older the gene, the higher the expression and the broader the expression i.e., high abundance and breadth of the gene, whereas the younger the gene, the lower the expression and the more specific the expression, i.e., the lower the abundance and the narrower the breadth of the gene. Indeed, this study used GEA and GEB together with PS (Figure 1). Therefore, purifying selection cannot be a major driving force for gene expression divergences. Furthermore, our results do not support neutral selection (Ks) as the major driving force for the evolution of divergences in gene expression, since Ks and gene expression had almost no correlation in this study (Figure 3, Figure 4 and Figures S2–S9). On the other hand, based on how new genes were associated with a high incidence of positive selection (Figure 1 and Figure 3), it is reasonable to assume that positive selection is the driving force for specific and low gene expression, while purifying selection is the stabilizing force for maintaining high GEA and large GEB, supporting positive selection as a driver for the origin and evolution of *Arabidopsis* genes.

A major concern is that we hypothesized that all ω > 1 loci were positively selected in this analysis. At first, this points to an overestimation of positive selection. However, strong purifying selection can disguise positive selection. In addition, positive selection goes through decay and can degenerate [73,74,75]. In fact, we showed that the frequency of ω > 1 loci detected in orthologous gene pairs within *A. thaliana* ecotypes, between species and between genera were decreased from 630/8005 to 341/20,729 and 94/18,056, respectively. Furthermore, the anti-correlation of positive selection with gene expression, inclusion of the remaining loci from the *Arabidopsis* genome (27,206 loci) or the development of more sensitive approaches that can detect low expression or highly specific gene expression, might identify more positively selected loci relevant to gene expression divergences. In addition, even in strong negatively selected loci, not every amino acid position is selected by purifying selection or neutral selection, as positively selected amino acid sites are likely much more frequent than positively selected gene loci [74,76]. Together, loci with positive selection might be rather widespread in the *Arabidopsis* genome [36], and positive selection is likely a force to refine gene expression divergences during evolution. Our study does not support the idea that natural selection is the major mechanism for trait evolution while neutral selection is the guiding principle for molecular evolution [39,77,78,79,80]; rather, it provides the molecular basis for Darwinian theory, thus reconciling natural selection and neutral selection at the molecular level [38,39]. Our study thus provides a foundation to study the congruence of divergences in protein sequences and gene expression, causing phenotypic diversification, and thus opens a door to broadly identify the molecular mechanism of natural selection on phenotypic adaptations in a variety of organisms [3,16].

More often, gene expression analysis is measured with microarrays, EST or RNA-seq. The gene expression level was affected by many factors, especially in multicellular organisms, such as gene length, introns, 5′-UTR and codon usage [71,81,82]. Thus, we often observe that highly expressed genes have less of an effect than lowly expressed genes, or that specifically expressed genes tend to have shorter introns. In this study, we did not consider these factors. Therefore, the next step should include these factors in these analyses.

## 4. Conclusions

Understanding the dynamic mechanisms underlying the evolution of gene expression is a fundamental challenge in evolutionary biology. In this study, a comprehensive analysis was conducted on the correlations between two key gene expression parameters—gene expression abundance (GEA) and gene expression breadth (GEB)—with gene age (as determined by phylostratum analysis) and evolutionary rates (Ka, Ks and ω), using genome-scale datasets encompassing intergenus, interspecies and intraspecies comparisons of *A. thaliana*. Our findings revealed that GEA is significantly negatively correlated with both phylostratum and evolutionary rates. Specifically, genes with higher expression levels tend to be evolutionarily older, indicating that highly expressed genes are more evolutionarily conserved, likely due to stronger purifying selection acting to preserve essential cellular functions. Similarly, GEB was negatively correlated with evolutionary rates, suggesting that genes with widespread functionality are also subject to stronger purifying selection. However, genes with lower GEA and narrower GEB have higher evolutionary rates, suggesting that these genes may be subject to positive selection, allowing for adaptive changes in response to environmental pressures or developmental needs. This pattern indicates that positive selection contributes to the origin and evolution of genes in *A. thaliana*, particularly those involved in specialized functions or rapid adaptation, thus supporting the Darwinian theory at molecular levels.

## 5. Materials and Methods

### 5.1. Data Mining

In the TAIR10 release (The *Arabidopsis* Information Resource, http://www.arabidopsis.org/, accessed on 20 December 2020) for *A. thaliana*, there are 41,671 gene models that include both representative gene models and their splice variants. This study retained only the representative variant but removed the chloroplast and mitochondrial genes, as well as other splice variants, to obtain 33,323 unique gene models. This study then removed transposable elements, pseudogenes, microRNAs, small RNAs, rRNAs, tRNAs and other non-coding RNAs to obtain 27,206 unique gene models that can encode protein peptides (Table S1). The protein and CDS sequences of *A. thaliana* (Ws-0), *A. thaliana* (Col-0), *A. lyrata* and *C. rubella* were downloaded from the 1001 Genomes Project (http://www.1001genomes.org/, accessed on 5 January 2021), TAIR10 and Phytozome v13 (http://phytozome.jgi.doe.gov/, accessed on 5 March 2021), respectively. All but one member of duplicated genes were removed, so that only the best matched pairs of orthologs in the two species were analyzed. Gaut’s microarray data [36] as well as microarray data from sperm, egg, central cells, synergid cells, pollen and seedlings were obtained and processed as previously described [29,52]. AtPANP [29] was used to call for the presence (*p*)/absence (A) of gene expression for single-celled samples. In the case of samples with two cell types (e.g., sperm and vegetative cells in pollen) and multicellular seedlings, we utilized the MAS5 tool for preprocessing and analysis of gene expression data [36,47,52]. The microarray expression signals were either derived from dCHIPs or RMA. RNA-Seq datasets for pollen and seedlings were from previous reports, and genes with at least 1 RPM (reads per million) were considered reliably expressed [45]. The gene models and loci associations with ESTs and cDNAs were obtained from collections at the TAIR website and deemed to be reliable. Phylostratum (PS) was directly assigned to all *Arabidopsis* genes to a PS (from PS 1 to PS 13) to coalesce a gene clade based on the origin of a defined protein domain in organisms during evolution, as shown in [23] and Table S1, followed by its correlation analysis with gene expression divergences.

### 5.2. Calculation of Ka/Ks (ω) Values

This study first identified orthologous gene pairs between species (*A. thaliana* (Col-0) and *A. lyrata*), between genera (*A. thaliana* (Col-0) and *C. rubella*) and within species (*A. thaliana* (Ws-0) and *A. thaliana* (Col-0)) using BLAST 2.2.29+ (https://ftp.ncbi.nlm.nih.gov/blast/executables/blast+/2.2.29/, accessed on 8 March 2021) by carrying out an all-blast (to)-all protein matched with *E*-value = 1 × 10^−5^ and identity ≥ 90% as cut-offs. Then, orthologous gene sequences were arranged for further analysis by Perl scripts. The ParaAT1.0 [83] (https://ngdc.cncb.ac.cn/tools/paraat?lang=zh, accessed on 20 March 2021) program was used to align the coding regions of genes and compute nonsynonymous (Ka) and synonymous (Ks) substitution rates and selective constraint ω (Ka/Ks) using the YN method with KaKs_Calculator2.0 [84] (http://sourceforge.net/projects/kakscalculator2/, accessed on 20 March 2021). Analysis of Ka and Ks is widely used to distinguish between fast- and slow-evolving protein-coding genes, or variable and conservative protein-coding genes. This study removed Ka > 1 and Ks > 5, as well as Ka or Ks with 0 and n.a. by manual adjustment, then derived data based on Ks (α = 0.05). Finally, this study analyzed (18,056/27,206), (20,729/27,206) and (8005/27,206) of all the unique coding sequences between genera, between species and within species, respectively (Tables S1 and S8).

### 5.3. Data Grouping and Statistical Analysis for Gene Expression Abundance and Breadth

To analyze GEA, loci were pooled according to gene expression amount after ranking their expression level. For ESTs and cDNAs, groups were pooled by the number of ESTs or cDNAs per locus, while for microarray (Gaut’s microarrays) [36] and RNA-Seq data, groups were defined by a non-overlapping sliding window of 100 loci according to expression abundance (GEA), as each such data point was correspondent to an average of 100 loci. To estimate gene expression breadth (GEB), loci with the number of “present call” in each sample were counted. Specifically, for EST or cDNA datasets, the “present call” meant that the locus had at least one EST or cDNA in the collections and for microarray or RNA-Seq datasets, the “present call” meant that each locus had a signal beyond a defined signal threshold (details shown in the paragraph of “data mining”). Graphic and statistical analyses were performed using either GraphPad Prism version 5.0 for Windows (GraphPad Software, San Diego, CA, USA, www.graphpad.com, accessed on 25 March 2019) or Microsoft Excel 2010 (Microsoft, https://www.microsoft.com/en-us/microsoft-365/previous-versions/office-2010, accessed on 25 April 2019).

### 5.4. Normal Approximations of Ks

To quantify what extent the observed data “reasonably approximated” a normal distribution, this study divided both the observed data (Ks) and randomly generated data with the same mean and standard deviation as the observed values into 40 equal quantile intervals. These intervals were determined solely based on mathematical equal-length partitions (independent of the distribution of those data), with the minimum and maximum values of the dataset serving as the starting and ending points. Subsequently, this study counted the number of observations within each interval and utilized these counts as the distribution statistical values of raw data for visualization and further chi-square goodness-of-fit testing. The null hypothesis is that the distribution is well-approximated by the normal distribution, so a small *p*-value would indicate deviance or poor approximation.

In order to further evaluate the genomic effect on protein evolution in *Arabidopsis* genes, this study analyzed the distribution and relationship of Ks and ω in the genome of *A. thaliana*. The locus from isologous gene pairs between species/genera and within species (At-Al/At-Cr/Ws-Col) were selected for analysis after cutting-off by Ks (α = 0.05) (Table S8). The code was performed with R version 4.0.1 (R Foundation for Statistical Computing, Vienna, Austria, https://cran.r-project.org/src/base/R-4/, accessed on 6 June 2021) can be found in “normal.test.r”, and was used to test the data out on to imitate plots.

Scripts for normal.test.r:
  # Takes a numeric vector x and number of partitions k.
  # Tests to see if x is reasonably approximately normal.
  # Generates a vector of same length as x using normal distribution with mean(x) and sd(x)
  # Bins both vectors k partitions.
  # Uses chi-sq test to see if counts are similar enough.
  norm.test = function(x, k){
  x = na.omit(x)
  mean.x = mean(x)
  sd.x = sd(x)
  norms = rnorm(length(x), mean.x, sd.x)
  breaks = seq(min(norms), max(norms), l=k+1)
  breaks [1] = −Inf
  breaks [2] = Inf
  normcounts = hist(norms, breaks, plot=FALSE)$counts
  xcounts = hist(x, breaks, plot=FALSE)$counts
  return(chisq.test(xcounts, normcounts))
  }
  atal = read.csv(“./S8.LociwithAt-Al.csv”, header=TRUE, skip=3)
  katal = atal$Ks
  watal = atal$X.
  norm.test(katal, 45) # Figure S1A (should be “reasonably well-approximated”)
  atcr = read.csv(“./S8.LociwithAt-Cr.csv”, header=TRUE, skip=2)
  katcr = atcr$Ks
  watcr = atcr$X.
  norm.test(katcr, 45) # Figure S1C (should NOT be “reasonably well-approximated”)
  Scripts for approximated normal distribution (Table S8):
  >norm.test(katal, 40) # Figure S1A (should be “reasonably well-approximated”)
       Pearson’s Chi-squared test
  data: xcounts and normcounts
  *X-squared = 1257.143, df = 1184, p-value = 0.06853*
  >norm.test(katcr, 40) # Figure S1C (should NOT be “reasonably well-approximated”)
       Pearson’s Chi-squared test
  data: xcounts and normcounts
  *X-squared = 1240, df = 1116, p-value = 0.005428*
  >norm.test(katal, 30) # Figure S1A (should be “reasonably well-approximated”)
       Pearson’s Chi-squared test
  data: xcounts and normcounts
  *X-squared = 720, df = 696, p-value = 0.2566*
  >norm.test(katcr, 30) # Figure S1C (should be “reasonably well-approximated”)
       Pearson’s Chi-squared test
  data: xcounts and normcounts
  *X-squared = 720, df = 696, p-value = 0.2566*

### 5.5. Correlation Grouping of Gene Expression Divergences and Sequence Divergences

This study only observed weak marginal correlations between GEA (gene expression divergences derived from RNA-Seq and microarrays, Tables S12 and S13), Ks and ω, pairwise. These weak correlations were shown in the “$old.cor” correlation matrices (x = Ks, y = ω, and z = log(GEA) in the matrix). This study then attempted to consider the correlation between E(Ks|GEA) and E(ω|GEA), or equivalently, the correlation after averaging out noise of Ks and ω that is unrelated to GEA. This study empirically estimated E(Ks|GEA) (or E(ω|GEA)) by averaging 100 Ks (or ω) values at loci having the closest gene expression values to the expression of a given locus (Tables S12J and S13J). Note that a high Pearson correlation between E(Ks|GEA) and E(ω|GEA) would indicate that Ks and ω are related to GEA in a similar way, or GEA has similar effects on Ks and ω.

This study ran simulations to demonstrate the above procedure with randomly generated vectors X, Y and Z abiding by X = f(Z) + noise and Y = g(Z) + noise, meaning that X and Y are both functions of Z plus some noise. The marginal correlation between X and Y depends on the noise level as well as the actual forms off() and g(). When the noise level is high, the relationship between x and y (or the relationship between f() and g()) would be buried under the noise, and a weak marginal correlation between x and y is likely to be observed. The correlation between E(X|Z) and E(Y|Z) is however robust to this noise, motivating us to use above procedure for processing GEA, Ks and ω. This study particularly took f(Z) = Z and g(Z) = Z with Z being normal to generate the simulation data and estimated E(X|Z = z) and E(Y|Z = z) by averaging the 100 nearest observations for x, y and z, grouped by z. Correlation matrices before and after this data processing are presented below in “$old.cor” and “$new.cor,” respectively.

R code (test.12 for Table S12J and test.13 for Table S13J):
  >test.s12=test_sim(s12$Ks,s12$X.,s12$Log10.RPMs, Ks)
  $old.cor
  xyz
  x1.00000000-0.2074137-0.08300841
  y-0.207413711.0000000-0.23241716
  z-0.08300841-0.23241721.00000000
  $new.cor
  avg.rank.xavg.rank.yavg.rank.z
  avg.rank.x1.0000000-0.25292290.1087751
  avg.rank.y-0.25292291.0000000-0.3475637
  avg.rank.z0.1087751-0.34756371.0000000
  >test.s13=test_sim(s13$X.1,s13$X.2,s13$X.4, Ks)
  $old.cor
  xyz
  x1.00000000-0.2115729-0.07317154
  y-0.211572921.0000000-0.33954236
  z-0.07317154-0.33954241.00000000
  $new.cor
  avg.rank.xavg.rank.yavg.rank.z
  avg.rank.x1.00000000.3940529-0.4705550
  avg.rank.y0.39405291.0000000-0.9003214
  avg.rank.z-0.4705550-0.90032141.0000000

### 5.6. Permutation Tests for Significant Differences in Correlations of ω and GEA Compared to Ks and GEA

To justify difference in correlations of ω and GEA compared to Ks and GEA, this study performed permutation tests to compare the differences between cor(ω, GEA) and cor(Ks, GEA). This study grouped observed GEA as shown in Tables S10–S20 and S21J (Figure 3A,C,E,G, Figures S3A,C,E,G and S5A,C,E,G). For each given dataset, there were n grouped observations. Under the null hypothesis, for a given value of GEA, each of the corresponding (ω, GEA) pair and (Ks, GEA) pair was equally likely to appear in either the observed n (ω, GEA) pairs or the observed n (Ks, GEA) pairs. Thus, there were a total of 2^n^ possible permutations of the n (ω, GEA) pairs and n (Ks, GEA) pairs. If 2^n^ ≤ 100,000, this study enumerated all possibilities and if 2^n^ > 100,000, we took a random sample for computational efficiency. This study then took the difference between cor(ω, GEA) and cor(Ks, GEA) as our test statistic. The resulting *p*-value is the probability that the enumerated differences are at least as extreme as the observed difference in correlations.

R code (given x= ω, y = Ks and z = GEA) for permutation tests for significant differences in correlations:
  perm.test = function(x, y, z){
  if(length(x) != length(y) || length(x) != length(z)){
  stop(“x, y, and z must be of equal length”)
  }
  if (2^(length(x)) <= 100000){
  allcombs = expand.grid(rep(list(c(TRUE, FALSE)), length(x)))} else {
  allcombs = t(replicate(100000, sample(c(TRUE, FALSE), length(x), replace=TRUE)))
  }
       allcors = apply(allcombs, 1, function(idx){cor(c(x[idx], y[!idx]), z)})
       alldiffs = allcors-rev(allcors)
       xcor = cor(x, z)
       ycor = cor(y, z)
       mydiff = xcor-ycor
       hist(alldiffs)
       abline(v=mydiff)
       if (mydiff>=0){
  return(c(mean((xcor-ycor) <= alldiffs))) } else {
  return(c(mean((xcor-ycor) >= alldiffs)))
  }
  wg.kg.perms = mapply(perm.test, x=ω, y=Ks , z=GEA)
  cor.sig = data.frame(wg.cor, wg.test.p, ifelse(xz.test.p<0.05, “Yes”, “No”),
  kg.cor, kg.test.p, ifelse(yz.test.p<0.05, “Yes”, “No”),
  wg.kg.perms, ifelse(xz.yz.perms<0.05, “Yes”, “No”))
  names(cor.sig) = c(“Cor.ω.GEA”, “p-value”, “Below 0.05?”,
  “Cor.Ks.GEA”, “p-value”, “Below 0.05?”,
  “perms.p-value”, “Below 0.05?”)
  rownames(cor.sig) = 10:21

### 5.7. GO and KEGG Enrichment Analysis

GO analysis was performed using the “GO Enrichment” function in TBtools v2.142 [63], and KEGG enrichment analysis was performed using the “KEGG Enrichment” function [63]. To filter for enriched categories or pathways, a significance threshold of *p*-value  <  0.05 and q-value  <  0.05 was applied, and the degree of enrichment was represented using the - log10 transformed *p*-value. The top 10 enriched results were visualized for all three modules of GO enrichment analysis which included biological processes, cellular components and molecular functions, and KEGG analysis using the “Enrichment Bar Plot” function in TBtools v2.142 [63].

## Figures and Tables

**Table 1:** Functional differences between gene age and gene expression patterns supported by mutant analysis in *Arabidopsis*.

Locus	Ka	Ks	ω	PS	EST	cDNA	RNA-Seq	Microarrays	Mutant
AT2G13560	0.0007	0.0641	0.0108	1	93	3	161.28	972.10	*nad-me1* [58]
AT2G33210	0.0008	0.0581	0.0132	1	34	3	51.22	1153.88	*hsp60-2-1* [59]
AT5G02870	0.0021	0.1137	0.0181	1	843	10	315.57	4365.06	*rpl4d* [60]
AT3G18980	0.0021	0.0097	0.2203	11	44	7	18.88	n.a.	*ein2* [61]
AT2G28240	0.0043	0.0035	1.2273	11	10	2	16.14	n.a.	*mom2-2* [62]

Note: n.a., not available.

## Data Availability

The original contributions presented in the study are included in the article/Supplementary Materials, and further inquiries can be directed to the corresponding authors.

## Supplementary Materials

The following supporting information can be downloaded at: https://www.mdpi.com/article/10.3390/ijms252413710/s1.
