# Supplementary material for: Fixation of Expression Divergences by Natural Selection in Arabidopsis Coding Genes
Source: Int J Mol Sci. 2024 Dec 22;25(24):13710. doi: 10.3390/ijms252413710 (PMC11678068; doi:10.3390/ijms252413710)
Supplement: Supplementary file 1 [file ijms-25-13710-s001.zip › Supplementary Figures and legends-R2.pdf]

Supplementary Figures and Legends:

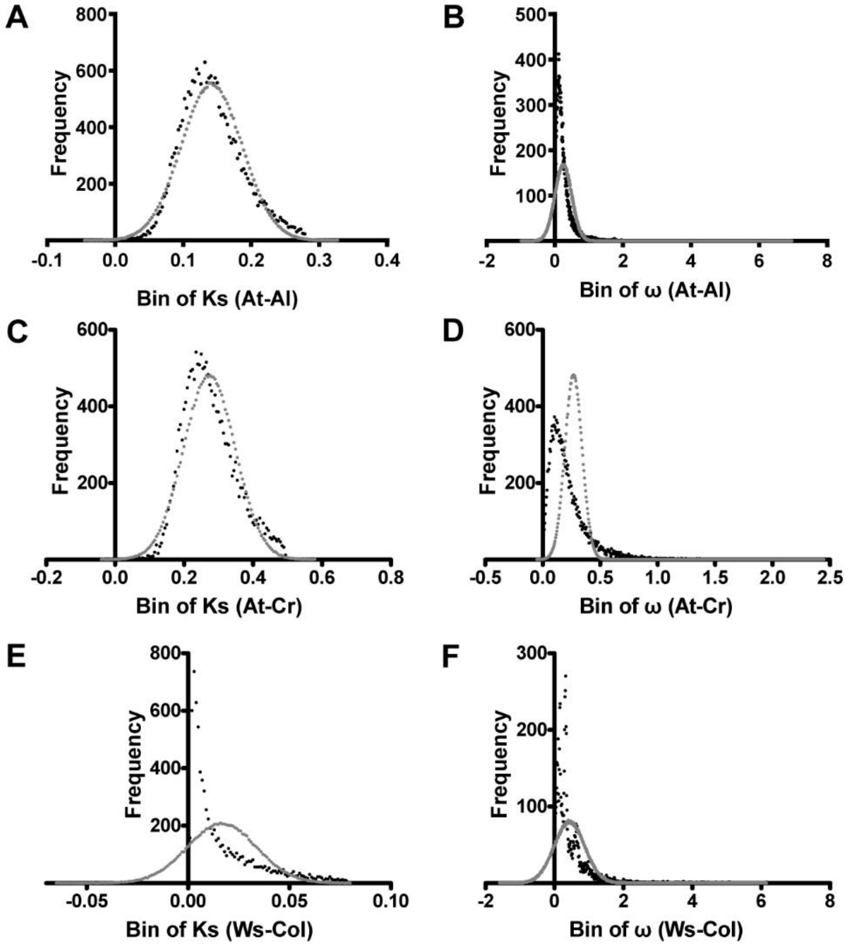

Fig. S1. Frequency distribution of Ks and Ka/Ks (ω) in *Arabidopsis* genome. Close to normal distribution of Ks in loci derived from orthologous gene pairs between species and between genera was observed (A and C)(methods), but skewed distribution of Ks in loci derived from orthologous gene pairs within species was observed (E). Skewed distribution for ω in loci derived from orthologous gene pairs between species, between genera and within species was observed respectively (B, D and F). Curves with dark color represent the frequency distribution of Ks and ω and curves with the light color for the theoretical line based on 1000 randomly generated values using NORMINV(RAND(), μ, σ).

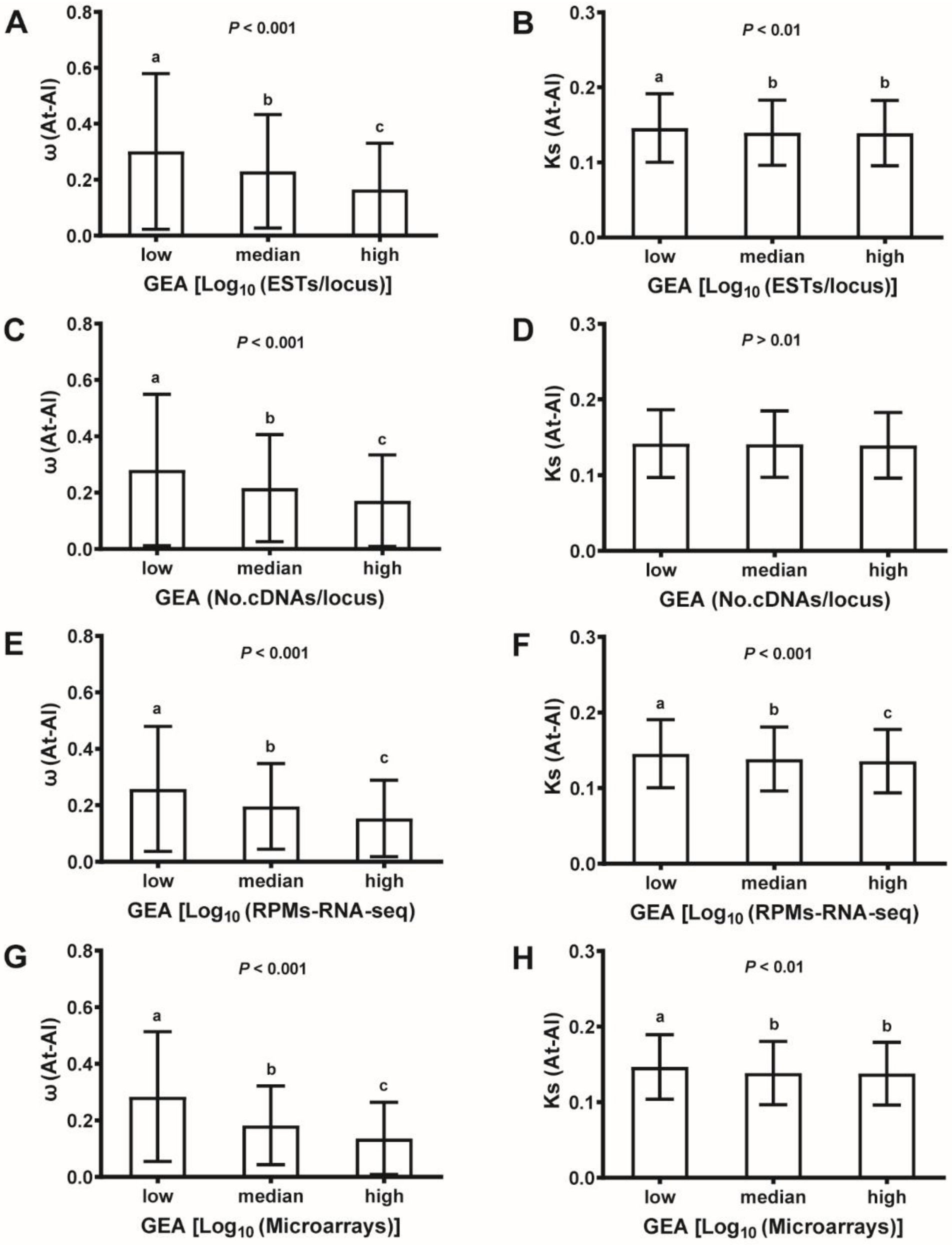

Fig. S2. Gene expression abundance (GEA) was strongly anti-correlated with selective constraint (ω) but weakly correlated with Ks derived from orthologous gene pairs between *A. thaliana* and *A.lyrata* (interspecies). GEA was divided into low, medium and high levels and then correlated with ω and Ks. ESTs/locus was strongly anti-correlated with ω (A) but weakly correlated with Ks (B). cDNAs/locus was strongly anti-correlated with ω (C) but weakly correlated with Ks (D). Log_{10} (RPMs) from RNA-Seq of seedlings was strongly anti-correlated with ω (E) but weakly correlated with Ks (F). RPMs: reads per million. Log_{10} (microarray signals) from Gaut's microarrays was strongly anti-correlated with ω (G) but weakly correlated with Ks (H). Error bars are standard deviation.

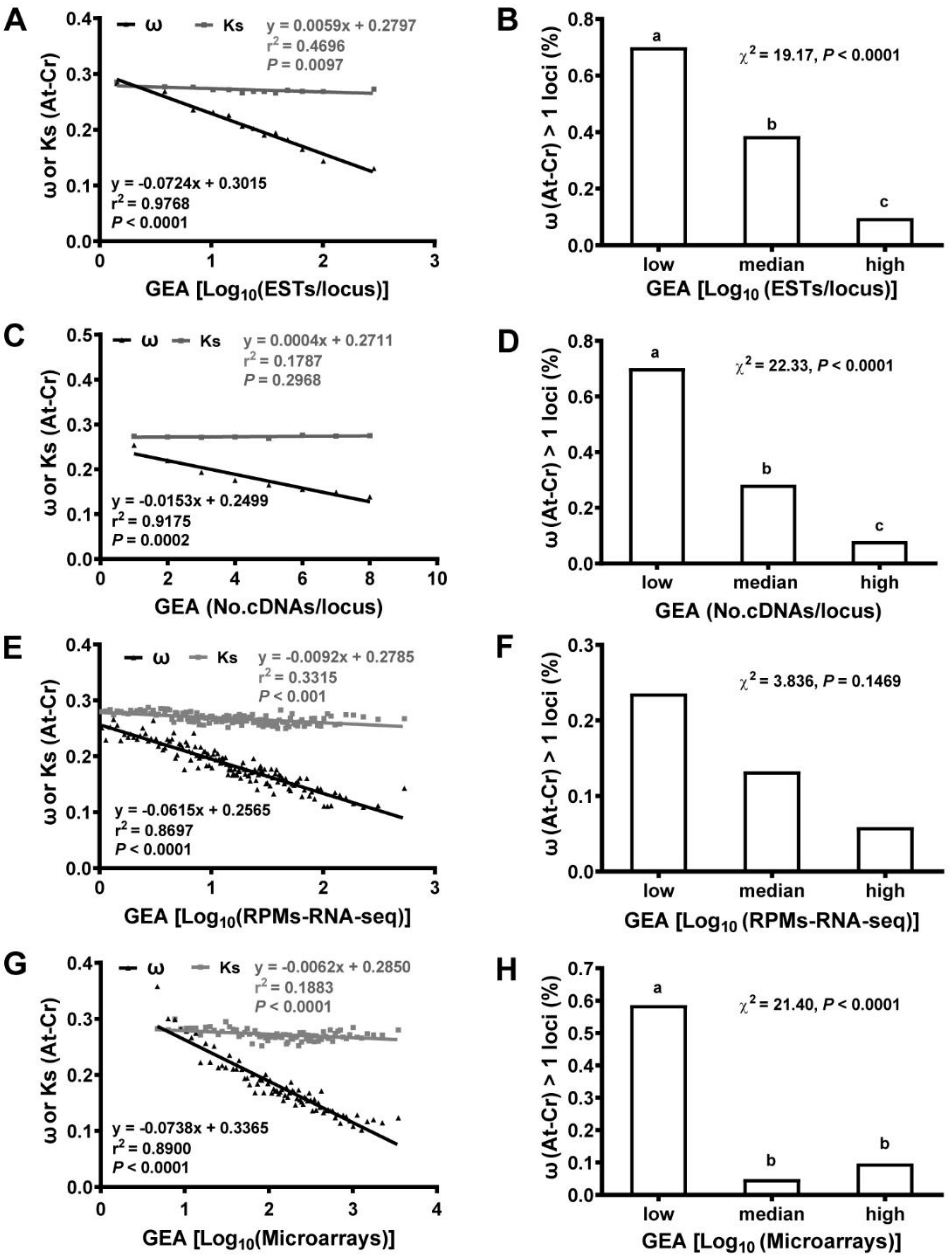

Fig. S3. Gene expression abundance (GEA) was anti-correlated with selective constraint (ω) and the incidence of ω > 1 loci (positive selection markers) derived from orthologous gene pairs between *A. thaliana* and *C. rubella* (intergenus). ESTs/locus was strongly anti-correlated with ω (A) and the incidence of positive selections (B) but weakly correlated with Ks (A). cDNAs/locus was strongly anti-correlated with ω (C) and the incidence of positive selections (D), but weakly correlated with Ks (C). Log_{10} (RPMs) from RNA-Seq of seedlings was anti-correlated with ω (E) and the incidence of positive selections (F), but not with Ks (E). RPMs: reads per million. Log_{10} (microarray signals) from Gaut's experiments was strongly anti-correlated with ω (G) and the incidence of positive selections (H), but weakly correlated with Ks (G).

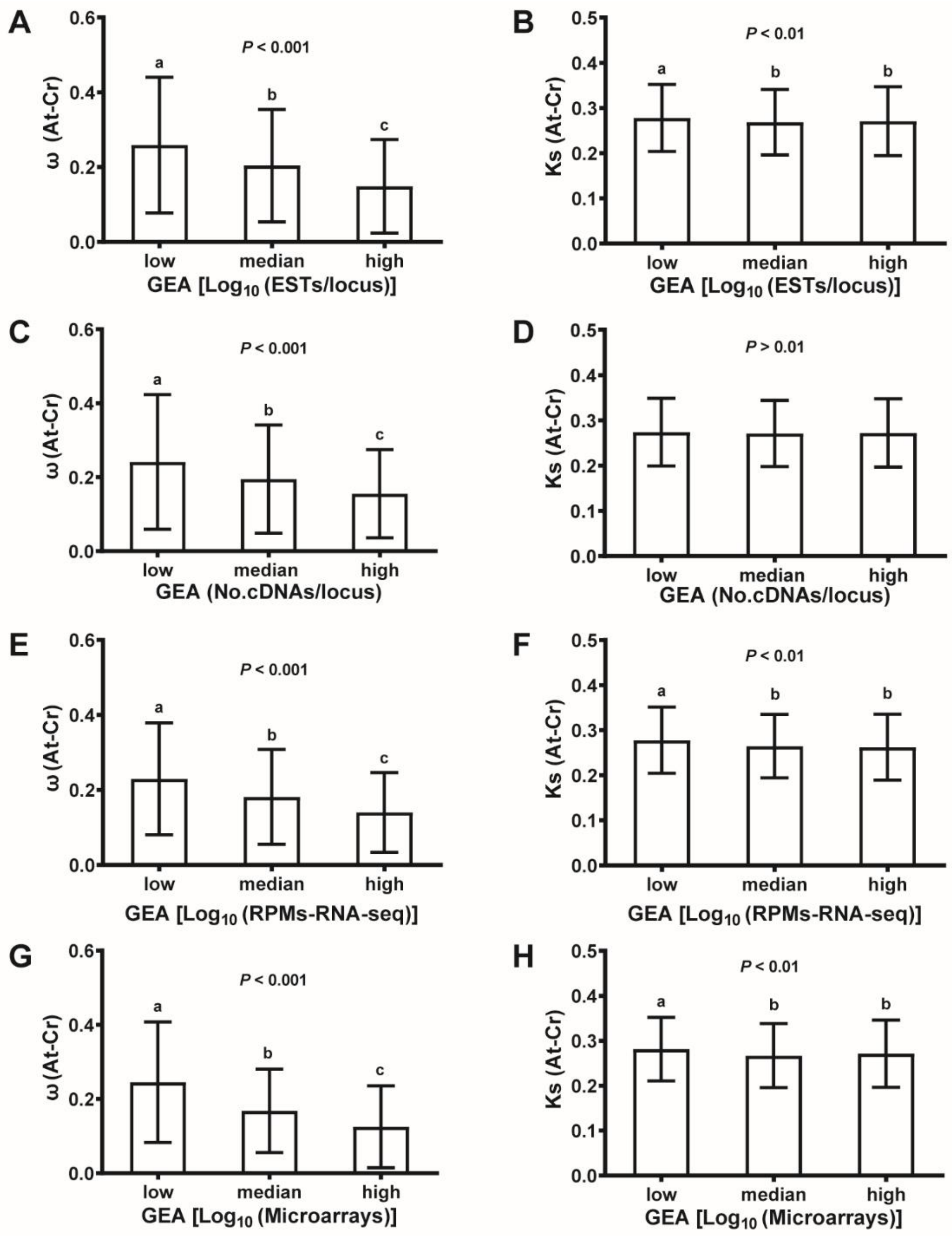

Fig. S4. Gene expression abundance (GEA) was strongly anti-correlated with selective constraint (ω) but weakly correlated with Ks derived from orthologous gene pairs between *A. thaliana* and *C. rubella* (intergenus). GEA was divided into low, medium and high levels and then correlated with ω and Ks. ESTs/locus was strongly anti-correlated with ω (A) but weakly correlated with Ks (B). cDNAs/locus was strongly anti-correlated with ω (C) but weakly correlated with Ks (D). Log_{10} (RPMs) from RNA-Seq of seedlings was strongly anti-correlated with ω (E) but weakly correlated with Ks (F). RPMs: reads per million. Log_{10} (microarray signals) from Gaut's experiments was strongly anti-correlated with ω (G) but weakly correlated with Ks (H). Error bars are standard deviation.

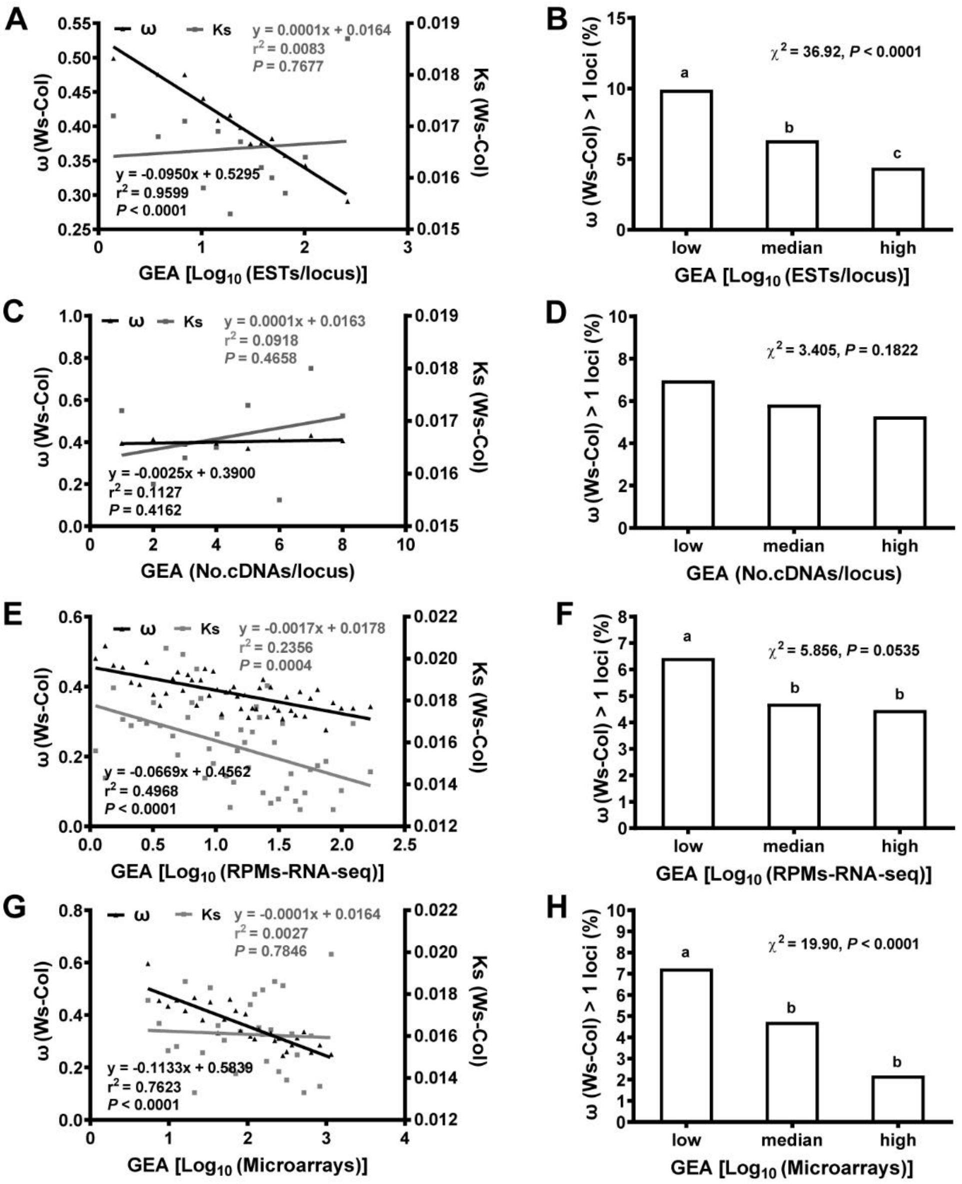

Fig. S5. Gene expression abundance (GEA) was consistently anti-correlated with the incidence of $\omega > 1$ loci but less consistently correlated with selective constraint (ω) and Ks from orthologous gene pairs between Arabidopsis Ws and Col. (A) ESTs/locus was strongly anti-correlated with ω but weakly correlated with Ks. (B) ESTs/locus was strongly anti-correlated with the incidence of positive selections (ω > 1 loci). (C) cDNAs/locus was negatively correlated with ω but positively correlated with Ks. (D) cDNAs/locus was strongly anti-correlated with the incidence of ω > 1 loci. (E) Log_{10} (RPMs) from RNA-Seq of seedlings was anti-correlated with ω and so was Ks. RPMs: reads per million. (F) Log_{10} (RPMs) from RNA-Seq of seedlings was strongly anti-correlated with the incidence of ω > 1 loci loci in 5 groups sorted by expression amount. (G) Log_{10} (microarray signals) from Gaut's experiments was strongly anti-correlated with ω but not Ks. RPMs: reads per million.

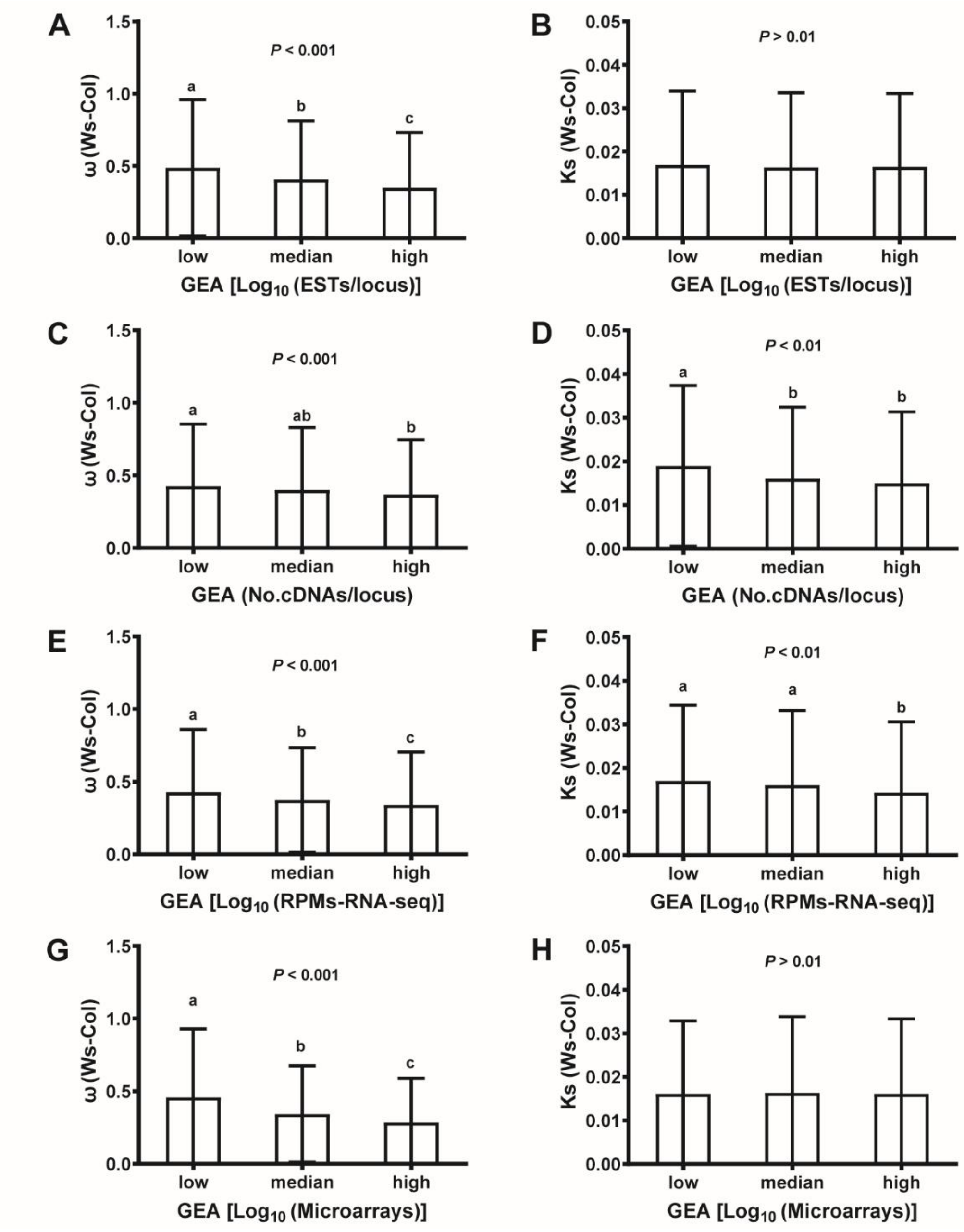

Fig. S6. Gene expression abundance (GEA) was strongly anti-correlated with selective constraint (ω) but weakly correlated with Ks derived from orthologous gene pairs between Arabidopsis Ws and Col. GEA was divided into low, medium and high levels and then correlated with ω and Ks. ESTs/locus was strongly anti-correlated with ω (A) but not Ks (B). cDNAs/locus was strongly anti-correlated with ω (C) but weakly correlated with Ks (D). Log_{10} (RPMs) from RNA-Seq of seedlings was strongly anti-correlated with ω (E) but weakly correlated with Ks (F). RPMs: reads per million. Log_{10} (microarray signals) from Gaut's experiments was strongly anti-correlated with ω (G) but weakly correlated with Ks (H). Error bars are standard deviation (E, F).

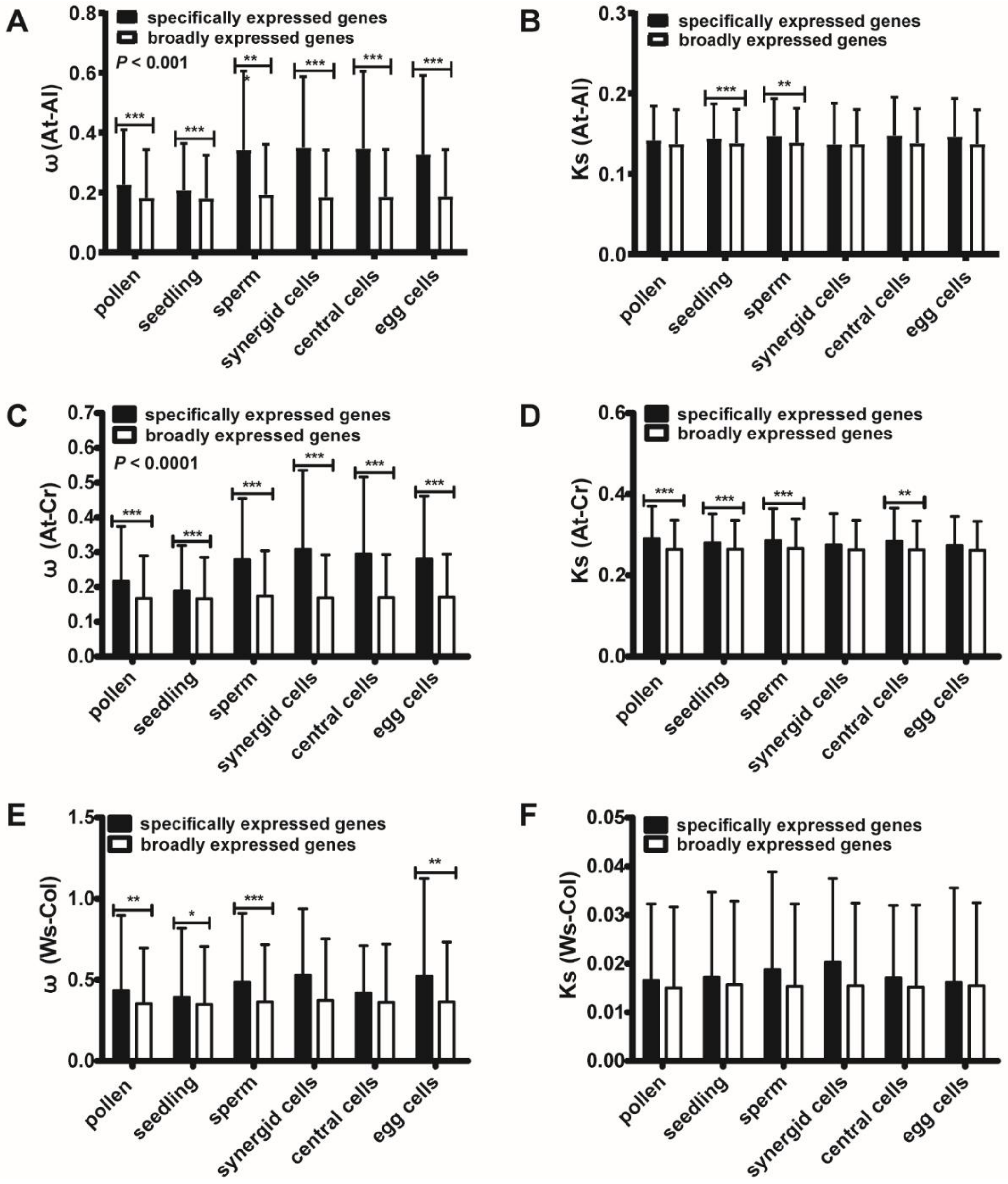

Fig. S7. Genes with specific expression had much higher ω but not much higher Ks. Genes expressed in one sample (black bars) had higher ω than genes that were expressed more than one samples (unfilled bars) (A, C and E). Genes expressed in one sample (black bars) only occasionally had higher Ks than genes that were expressed (unfilled bars) more than one samples (B, D and F). "***", "**" and "*" indicates significant difference at P < 0.001, 0.01 and 0.05 respectively. Error bars are standard deviation.

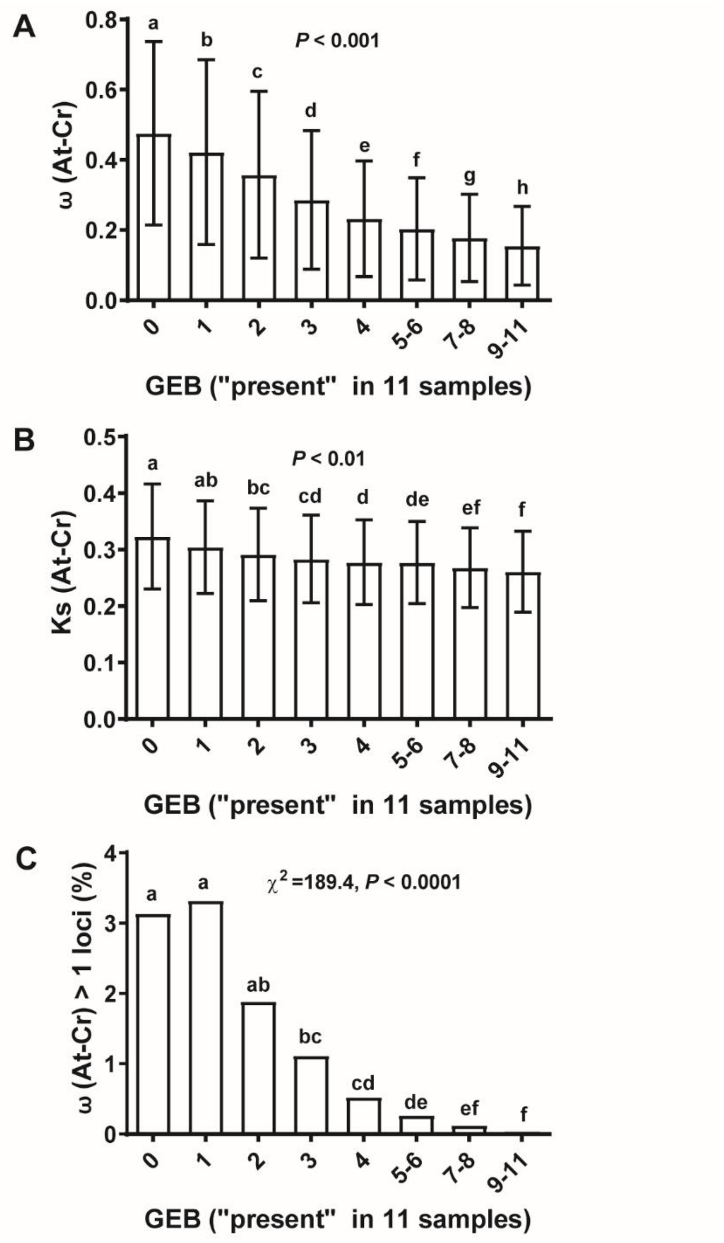

Fig. S8. Gene expression breadth (GEB) was strongly anti-correlated with selective constraint (ω) and the incidence of ω > 1 loci (positive selection markers) derived from orthologous gene pairs between *A. thaliana* and *C. rubella* (intergenus). (A) GEB was strongly anti-correlated with ω. a to h indicates a significant level at $P < 0.001$ for ω between any adjacent groups with differential GEB. Error bars are standard deviation. (B) GEB was only minimally anti-correlated with Ks (neutral selection markers). a to f indicates a significant level at $P < 0.01$. There was no significant difference for Ks between any adjacent groups. Error bars are standard deviation. (C) GEB was anti-correlated with the incidence of ω > 1 loci (positive selection markers). Narrowly expressed genes had significantly higher incidence of ω > 1 loci than broadly expressed genes ($\chi^2 = 189.4$, $P < 0.0001$). Error bars are standard deviation (A, B).

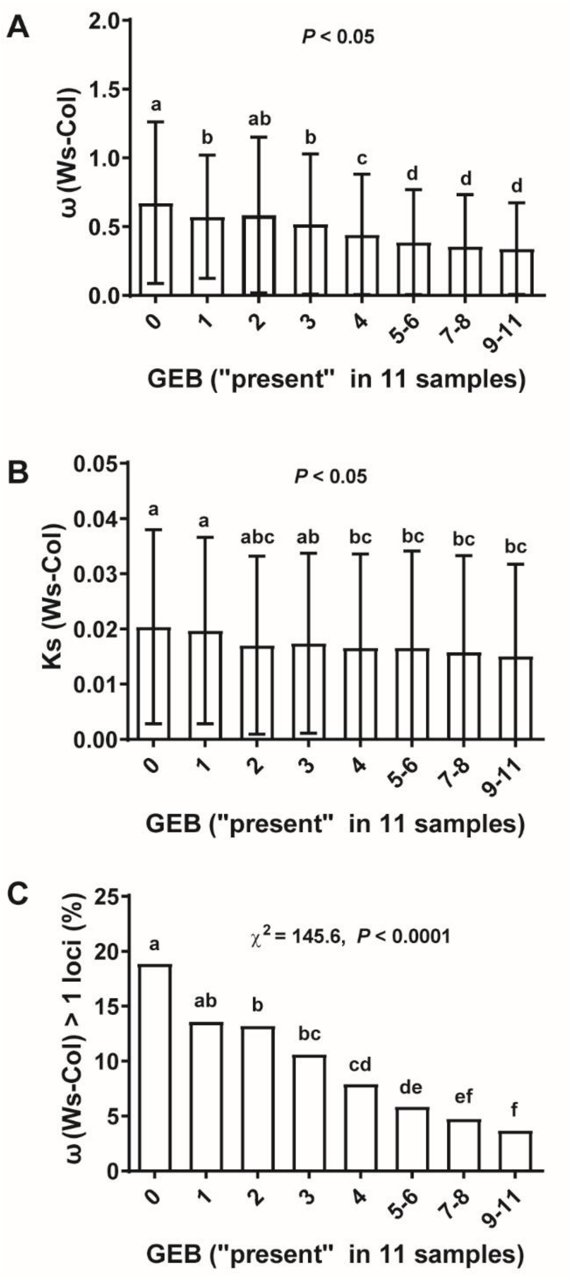

Fig. S9. Gene expression breadth (GEB) was anti-correlated with selective constraint (ω) and the incidence of ω > 1 loci (positive selection markers) derived from orthologous gene pairs between *A. thaliana* and *A.lyrata* (interspecies). (A) GEB was anti-correlated with ω in 4 groups. a to d indicates a significant level at P < 0.05 for ω between any adjacent groups with differential GEB. Error bars are standard deviation. (B) GEB was anti-correlated with Ks (neutral selection markers) in 3 groups. a to c indicates a significant level at P < 0.05. There was no significant difference for Ks in any adjacent groups. Error bars are standard deviation. (C) GEB was anti-correlated with the incidence of ω > 1 loci (positive selection markers) in 5 groups. Narrowly expressed genes had significantly higher incidence of ω > 1 loci than broadly expressed genes ($\chi^2 = 145.6$, P < 0.0001). Error bars are standard deviation (A, B).

Supplementary Table legends:
Table S1. Gene expression data and evolutionary proxies for *Arabidopsis* coding genes used in this study.
Table S2. Analysis of PS (phylostratum) in loci with GEA (No. ESTs).
Table S3. Analysis of PS (phylostratum) in loci with GEA (No. cDNAs).
Table S4. Analysis of PS in loci with gene expression abundance (GEA) [Log_{10} (RPMs)] from seedling RNA-Seq.
Table S5. Analysis of PS in loci with gene expression abundance (GEA) from Gaut's microarrays.
Table S6. Analysis of PS in loci with specific expression in microarrays of six samples.
Table S7. Analysis of PS in loci with gene expression breadth (GEB) in 11 samples.
Table S8. Selection of loci for evolutionary analysis of gene expression divergences.
Table S9. Analysis for Linkage disequilibrium.
Table S10. Analysis of Ks, ω and ω >1 (between species) in loci with GEA (No. ESTs).
Table S11. Analysis of Ks, ω and ω > 1 (between species) in loci with GEA (No. cDNAs).
Table S12. Analysis of Ks, ω and ω > 1 (between species) in loci with gene expression abundance (GEA) from seedling RNA-Seq.
Table S13. Analysis of Ks, ω and ω > 1 (between species) in loci with gene expression abundance (GEA) from Gaut's microarrays.
Table S14. Analysis of Ks, ω and ω > 1 (between genera) in loci with ESTs.
Table S15. Analysis of Ks, ω and ω > 1 (between genera) in loci with GEA (No. cDNAs).
Table S16. Analysis of Ks, ω and ω > 1 (between genera) in loci with gene expression abundance (GEA) from seedling RNA-Seq.
Table S17. Analysis of Ks, ω and ω > 1 (between genera) in loci with gene expression abundance (GEA) from Gaut's microarrays.
Table S18. Analysis of Ks, ω and ω > 1 (within species) in loci with GEA (No. ESTs).
Table S19. Analysis of Ks, ω and ω > 1 (within species) in loci with No. cDNAs.
Table S20. Analysis of Ks, ω and ω > 1 (within species) in loci with gene expression abundance (GEA) from seedling RNA-Seq.
Table S21. Analysis of Ks, ω and ω > 1 (within species) in loci with gene expression abundance (GEA) from Gaut's microarrays.
Table S22. Permutation tests for significant differences of the correlations of ω and GEA to the correlations of Ks and GEA in pairwise comparison.
Table S23. Analysis of ω, Ks (between species) in loci with specific expression (microarrays).
Table S24. Analysis of ω, Ks (between genera) in loci with specific expression (microarrays).
Table S25. Analysis of ω, Ks (within species) in loci with specific expression (microarrays) in six samples.
Table S26. Evolutionary analysis of female-enriched genes.
Table S27. Analysis of Ks, ω and ω > 1 (At-Al) in loci with gene expression breadth (GEB) in 11 samples.
Table S28. Analysis of Ks, ω and ω > 1 (At-Cr) in loci with gene expression breadth (GEB) in 11 samples.
Table S29. Analysis of Ks, ω and ω > 1 (Ws-Col) for loci with gene expression breadth (GEB) in 11 samples.
